# Labour induction with gestational hypertension: A great obstetric challenge

**DOI:** 10.12669/pjms.331.10386

**Published:** 2017

**Authors:** Meharun-Nissa Khaskheli, Shahla Baloch, Aneela Sheeba, Sarmad Baloch, Fahad Khan

**Affiliations:** 1 Dr. Meharun-Nissa Khaskheli, MBBS, FCPS. Associate Professor, Dept. of Obstetrics & Gynaecology, Liaquat University of Medical & Health Sciences, Jamshoro, Sindh, Pakistan; 2 Dr. Shahla Baloch, MBBS, DGO, FCPS. Associate Professor, Dept. of Obstetrics & Gynaecology Liaquat University of Medical & Health Sciences, Jamshoro, Sindh, Pakistan; 3 Dr. Aneela Sheeba, MBBS, DMRD, FCPS. Assistant Professor, Dept. of Radiology, Liaquat University of Medical & Health Sciences, Jamshoro, Sindh, Pakistan; 4 Dr. Sarmad Baloch, MBBS. House Officer, Medical Department, Liaquat University of Medical & Health Sciences, Jamshoro, Sindh, Pakistan; 5 Dr. Fahad Khan, MBBS. House Officer, Medical Department, Liaquat University of Medical & Health Sciences, Jamshoro, Sindh, Pakistan

**Keywords:** Gestational hypertension, Induction of labour, Maternal and fetal complications

## Abstract

**Objective:**

To observe the fetomaternal morbidity and mortality with induction of labour in pregnant women with gestational hypertension.

**Methods:**

The subjected study population included was 138 pregnant women with gestational hypertension. These pregnant women were between 34-40 weeks of gestational period in whom labour was induced, while the pregnant women who had labour induction for other reasons were excluded. These women were registered on the predesigned proforma. The data was collected and analyzed on SPSS version 21.

**Result:**

Out of the 138 cases, mean age of the women was 25.93±5.037, prim gravid women were 78(56.5%), gestational period in majority of these women 71(51.4%) varied between 35-38 weeks. The common presenting symptoms were oedema 119(86.23%), headache 90(65.21%). Labour induction in majority of the cases 81(58.7%) was carried with prosten pessary. The Caesarean section was needed in 39(28.3%) women in emergency due to maternal and fetal reasons or due to failed induction. Maternal complications were uncontrolled hypertension 23(16.7%), intensive care unit admission 21(15.2%), fits 15(10.9%), post partum haemorrhage 13(9.4%). Fetal complications were birth asphyxia 49(35.5%), neonatal intensive care unit admission 17(12.3%), neonatal death 14(10.1%).

**Conclusion:**

The emergency Caesarean section rate was quite high with induction of labour in pregnant women with gestational diabetes. The maternal morbidity as well as fetal morbidity and mortality rate was also high.

## INTRODUCTION

Pregnancy induced hypertension complicates about 10% of pregnancies, but there is a widespread geographical variation in its incidence. The incidence is higher in developing countries.^1^ Labour induction is an important option for the termination of pregnancy, when the progression of pregnancy increases the risks on the maternal health as well as on growing fetus. Labour induction with unfavorable cervix is done with prostaglandins, cervical Foleys catheter, mesoprostol then followed by amniotomy and augmentation with oxytocin, when cervix is favorable then induction with amniotomy and augmentation with oxytocin is carried out.^2^

Women with gestational hypertension or with mild pre-eclampsia at term, induction of labour is less costly than expected,^3^ but before taking the decision for labour induction women should be thoroughly evaluated regarding subsequent harmful effects of induction. Surprisingly the effectiveness for the induction of labour is not proven, so decision for induction of labour must be individualized.^4^,^5^ The safety and effectiveness of labour induction depends on the health of the woman and her baby, previous obstetrical history, appropriate time and method of induction and the availability of the birth capacity.

Labour induction in women with gestational hypertension who had completed 38 weeks pregnancy duration results in better outcome in comparison with those women in whom induction of labour is carried out at earlier gestation period. In these women there are increased risks for the babies like breathing problems, infections as well as neonatal intensive care unit admission.^6^ Despite a clinical benefit of induction of labour, long-term health-related quality of life(HR-Qol) is equal after the induction of labour and expectant management in women with gestational hypertension or pre-eclampsia beyond 36 weeks of gestation.^7^

Labour induction increases the chances of Caesarean section especially in primigravidas as cervix fails to soften and open. Many studies have unequivocally shown that the labour arrest and Caesarean delivery is high in the presence of an unripe cervix.^8^-^10^ It also requires careful monitoring during the process of labour by maintaining the progress with partographic record, intravenous line, continous electronic fetal monitoring and use of medication after birth to reduce the risk of haemorrhage.

There is a lack of clinical and practical evidence of immediate delivery and expectant management in the pregnant women with hypertensive disorders. The decision for termination of pregnancy in women with pregnancy induced hypertension and its associated consequences must be individualized considering its effects on the maternal health and fetal outcomes; there is a strong need of time to conduct such types of studies.

## METHODS

This is an observational study conducted at the Department of Obstetrics and Gynaecology unit IV, Liaquat University of Medical and Health Sciences Jamshoro for the period of one year from 1^st^ January 2015 to 31^st^ December 2015. During this study period 138 pregnant women were registered for induction of labour. They were admitted through Outpatient Department for their antenatal checkup and via Casuality Department in emergency with symptoms of severe pregnancy induced hypertension. Sample size was calculated by applying formula (N= (z) ^2^(pq)/e^2^) (Pregnancy induced hypertension complicates about 10% of pregnancies^1^, sample size was calculated by empirical means with confidential interval 95%). Sampling technique was Non probability convenient.

These women were registered on the predesigned proforma after taking informed written consent and taking approval from institutional ethical review committee. These women were between 15-36 years of age, primigravid as well as multigravid, their gestational period varied between 30-40 weeks. Presented clinical spectrum was oedema, headache, pain in epigastrium, blurring of vision. On clinical examination their blood pressure was recorded, symphysiofundal height was measured, fetal heart sounds were recorded. Relevant investigations such as complete blood picture, blood group, blood sugar level, screening of virology markers, liver function tests, renal function tests, ultrasonographic examination in all as well as Doppler ultrasound examination in required cases were done.

These women were managed with the departmental management protocol for pregnancy induced hypertension, pre-eclampsia, and eclampsia, when their condition was established; labour induction was decided with pelvic assessment, cervical condition, for fetal assessment cardiotocographic examination was carried. Appropriate mode for labour induction was decided after Bishop^s^ scoring and methods used were prostaglandin pessary, intracervical Foleys catheter and syntocinon infusion. Patients general condition, fetal condition and labour progress was vigilantly monitored with partographic record and fetal condition was monitored with cardiotocography. Immediate intervention steps were taken with any maternal or fetal problem according to the available institutional facilities along with multidisciplinary approach.

The data was collected and analyzed on IBM SPSS version 21, INC USA, 2012. The frequency and percentage was calculated for continuous variables, age was calculated with mean and standard deviation, Chi-Square statistical test was applied for qualitative type of analyses-Value <0.05 was considered significant.

## RESULTS

Out of total 138 women included in the study 29(21%)women were up to 20 years of age, 26(18.8%) women were 31 and above years of age, while 83(60.14%) women were between 21-30 years of age. The mean age with standard deviation was 25.93±5.037. The primigravid women with gestational hypertension with labour induction were more frequent 78(56.5%) in comparison with Para 3 and above 35(25.36%), majority of these women 71(51.4%) were having gestational period between 35-38 weeks; whereas 42(30.4%) women were having pregnancy duration between 30-34 weeks (P-Value .000). (Table-I).

**Table I:** Sociodemographic characteristics N=138.

S/No	Sociodemographic characteristics	No: of cases	Percentage	Chi-Square test	P-Value
1.	***Age in years***				
	Mean± SD: 25.93±5.037	29	21		
	a. upto 20	83	60.14		
	b. 21-30	26	18.8		
	c. 31 and above				
*2.*	***Parity:***				
	a. Primigravida	78	56.5		
	b. Para 1-2	25	18.1		
	c. para 3 and above	35	25.36		
3.	***Gestational period in weeks:***				
	a. 30-34	42	30.4		
	b. 35-38	71	51.4		
	c. 39 and above	25	18.1	49.388	0.000

Common presenting symptoms were oedema 119(86.23%), headache 90(65.21%), pain in epigastrium 78(56.52%), blurring of vision 65(47.1%). Cervical condition was favorable in 77(55.8%) women, and unfavorable in 61(44.2%) women. Labour induction was done with prostaglandin pessary in 81(58.7%) women, while other methods of induction of labour used were syntocinon infusion in 21 (15.2%)women, and intracervical Foleys catheter in 36(26.1%) women (P-Value 0.803). (Table-II).

**Table-II:** Symptomatology, Clinical findings, Methods of labour induction N=138.

Symptomatology, Clinical findings, Methods of labour induction	No. of cases	Percentage	Chi-Square test	P-Value
***1. Symptomatology***				
a. Headache	90	65.21		
b. Pain in epigastrium	78	56.52		
c. Blurring of vision	65	47.1		
d. oedema	119	86.23		
***2. Clinical findings: Bishops score***				
a. Favorable	77	55.79		
b. Unfavorable	61	44.20	4.754	.313
***3. Methods of labour Induction***				
i. Medical:				
a. Prostaglandin Pessary	81	58.7	4.562	.803
b. Syntocinon infusion	21	15.2		
ii. Mechanical				
a. Intracervical Foley’s catheter	36	26.1		

Labour induction outcome resulted in normal vaginal delivery in 89(64.49%) women, instrumental vaginal delivery in 10 (7.2%) women, while Caesarean section was performed totally in 39(28.3%) women, out of these failed induction comprises 12(30.76%) women and 27(69.23%) women had in emergency Caesarean section for maternal or fetal reasons(P-Value 0.126). Maternal complications observed were uncontrolled hypertension in 23(16.7%) cases, intensive care unit admission in 21(15.2%)cases, emergency anaesthesia complications in 17(12.3%) cases, fits in 15(10.9%) cases, postpartum haemorrhage in 13(9.4%) cases, perineal tear in 9(6.5%) cases and shoulder dystocia was seen in 2(1.4%) cases(P-Value 0.367). Fetal birth with good apgar score was seen in 45(32.60%) cases, while fetal complications observed were birth asphyxia in 49(35.5%) cases, intensive care neonatal unit admission in 17(12.3%) cases. Still birth in 13(9.4%) cases and early neonatal death was seen in 14(10.1%) cases (P-Value 0.090).(Table-III).

**Table-III:** Labour induction outcome and fetomaternal complications (N=138).

S/No	Labour induction outcome and fetomaternal complications	No of cases	Percentage	Chi-Square test	P-Value
1	***Labour induction outcome***				
	a. Normal vaginal delivery	89	64.49	7.193	0.126
	b. Instrumental vaginal delivery	10	7.2		
	c. Caesarean section	39	28.3		
2	***A: Maternal complications***				
	a. Uncontrolled hypertension	23	16.7		
	b. Intensive care unit admission	21	15.2		
	c. Emergency anaesthesia complications	17	12.3		
	d. Fits	15	10.9		
	e. Postpartum haemorrhage	13	9.4		
	f. Perineal tear	9	6.5		
	g. Shoulder dystocia	2	1.4	29.930	0.367
	***B. Fetal complications***				
	a. Birth asphyxia	49	35.5		
	b. Intensive care neonatal unit admission	17	12.3		
	c. Early neonatal death	14	10.1		
	d. Still birth	13	9.4	23.991	0.090

## DISCUSSION

Labour induction is carried out in 20% pregnancies in developed countries.^11^ It is an important option when continuation of the pregnancy increases the risk on the mother and baby, but the induction of labour itself increases the risks as well as there are chances of its failure. The safety and effectiveness of labor induction depends on the health of the woman and her baby. In this study most of the pregnant women 83 (60.14%) presented between 21-30 years of age and were prim gravid 78(56.5%), in comparison with Nigerian study^12^ wherein common age of the women were between 25-29 years (30.4%) and nulliparous women were 39.1%, this difference could be due to the early marriage and high frequency of hypertensive disorders in prim gravid women.

The gestational period was between 35-38 weeks in majority of the women 71(51.4%), this is tertiary care hospital receiving all the referred cases, these were the women with gestational hypertension and with associated problems, considering the health of the women and her baby labour induction was decided. Comparing with other studies^13^,^14^ pregnant women with gestational hypertension having induction of labour between 38-39 weeks leading to lowest chances for maternal and neonatal morbidity and mortality as well as lower in cost and decrease Caesarean section rate. Despite the lack of evidence that would justify intervention, many obstetricians induce labour in women at term with pregnancy-induced hypertension or preeclampsia. Such a policy may increase the risk of assisted vaginal delivery and caesarean section, thus generating additional morbidity and costs.^15^ Proper antenatal cares and hospitalization in case of need at appropriate time with careful fetomaternal monitoring will help in continuation of pregnancy till term and it will lead to improvement in maternal condition and fetal outcome.

Presenting complaints with gestational hypertension were oedema 119(86.23%), headache 90(65.21%), pain in epigastrium 78(56.52%), blurring of vision 65(47.1%), severity of the gestational hypertension is associated with retinopathy.^16^ In this study 15(10.9%) women had fits in comparison with other national study the frequency of eclampsia was 3/100.^17^ This difference could be due to various reasons like un-booked status, late referral, and non-availability of proper labour analgesics (Epidural). The severity of pregnancy induced hypertension is assessed with the extent of symptoms.^18^ Cervix was unfavorable in majority of the cases and labour induction was performed with prostaglandins in 81(58.7%) women, other methods were syntocinon infusion, intracervical foley, scatheter. These all are the recommended methods for the induction of labour.^19^ Labour induction outcome resulted in normal vaginal deliveries in 89(64.49%) women, instrumental vaginal deliveries in 10(7.2%) women. Caesarean section was performed in 39(28.3%) women due to failed induction or with maternal or fetal reasons, this is consistent with Zaiba Sher et al. study.^20^

In this study maternal morbidities and fetal morbidities as well as fetal mortality rate was higher. This can be due to the unbooked status, severity of the condition, and limited intensive care facilities for the new born, low birth weight, and poor fetal reserves. The clinical course of pregnancy induced hypertension is progressive as its severity increases the chances of fetomaternal complications that can only be stopped by delivery therefore the frequency of labour induction and related fetomaternal morbidity is high considering all these factors early detection of the risky cases and with timely, early appropriate management will overcome this.^21^,^22^

## CONCLUSION

Caesarean section rate was high due to failed induction and fetomaternal reasons in emergency. Maternal morbidity as well as fetal morbidity and mortality rate was high. Appropriate decision prior to induction of labour considering the condition of mother and fetus is very important. Vigilant labour monitoring, timely decision for intervention, and proper newborn care will help in decreasing morbidity and mortality.
